# Effect of spectral CT on tumor microvascular angiogenesis in renal cell carcinoma

**DOI:** 10.1186/s12885-021-08586-x

**Published:** 2021-07-30

**Authors:** Bei Zhang, Qiong Wu, Xiang Qiu, Xiaobo Ding, Jin Wang, Jing Li, Pengfei Sun, Xiaohan Hu

**Affiliations:** 1 Department of Radiology, First Hospital of Jilin University, No. 1, Xinmin Street, Changchun, Jilin Province China; 2 Department of Pathology, China-Japan Union Hospital, Jilin University, Changchun, China; 3 Department of Urology Surgery, First Hospital of Jilin University, Changchun, China

**Keywords:** Angiogenesis, Dual-energy CT, Renal cell carcinoma, Microvessel angiogenesis, Quantitative imaging

## Abstract

**Background:**

To examine the value of energetic-spectrum computed tomography (spectral CT) quantitative parameters in renal cell carcinoma (RCC) microvascular angiogenesis.

**Methods:**

The authors evaluated 32 patients with pathologically confirmed RCC who underwent triple-phase contrast-enhanced CT with spectral CT imaging mode from January 2017 to December 2019. Quantitative parameters include parameters derived from iodine concentration (IC) and water concentration (WC) of 120 keV monochromatic images. All specimens were evaluated including the microvascular density (MVD), microvascular area (MVA) and so on. The correlation between IC and WC (including average values and random values) with microvascular parameters were analyzed with Pearson or Spearman rank correlation coefficients.

**Results:**

The MVD of all tumors was 26.00 (15.00–43.75) vessels per field at × 400 magnification. The MVD of RCC correlated positively with the mean IC, mean WC, mean NWC, mean NIC, random IC, random NIC in renal cortical phase, WCD_1_, WCD_2_, NWCD_2_ and ICD_1_ (Spearman rank correlation coefficients, r range, 0.362–0.533; all *p* < 0.05). The MVA of all tumors was (16.16 ± 8.98) % per field at × 400 magnification. The MVA of RCC correlated positively with the mean IC, mean WC, mean NWC, mean NIC, random IC, random NIC in renal cortical, mean WC and mean NWC in renal parenchymal phase, WCD_1_, WCD_2_, WCD_3_, NWCD_2_, and NWCD_3_ (Pearson or Spearman rank correlation coefficients, r range, 0.357–0.576; all *p* < 0.05). Microvascular grading correlated positively with the mean NWC, mean NIC and random NIC in renal cortical phase, mean NWC in renal parenchymal phase, NWCD_2_, WCD_3_, NWCD_3_, NICD_2_ and NICD_3_ (Spearman rank correlation coefficients, r range, 0.367–0.520; all p < 0.05). As for tumor diameter (55.19 ± 19.15), μm, only NWCD_3_ was associated with it (Spearman rank correlation coefficients, r = 0.388; p < 0.05).

**Conclusions:**

ICD and WCD of spectral CT have a potential for evaluating RCC microvascular angiogenesis. MVD, MVA and microvascular grade showed moderate positive correlation with ICD and WCD. ICD displayed more relevant than that of WCD. The parameters of renal cortical phase were the best in three phases. NICD and NWCD manifested stronger correlation with microvascular parameters than that of ICD and WCD.

## Introduction

Renal cell carcinoma (RCC) is a kind of tumor originated from renal epithelial cells, accounting for more than 90% of renal cell carcinoma, consisting of 2% of adult malignancies [1, 2]. The incidence rate of RCC has increased in the past two decades [3]. Approximately, 20–30% of RCC patients will have distant metastasis during the follow-up period after curative surgery [3, 4]. However, the treatment of advanced RCC is still a challenge for clinicians, with a 5-year survival rate less than 20% [5]. Clear cell renal cell carcinoma (ccRCC) is the most common subtype, ccRCC accounts for almost 85% of all sorts of RCC [4, 5].

Due to the formation of new microvessels, the structure of microvessels in malignant tumors is different from that in normal tumors. In malignant tumors, microvessels are curved and organized more irregularly. In addition, their walls are more fragile due to the weak connection among endothelial cells, other pericyte layers and basement membrane [6]. These characteristics are related to the susceptibility of tumor invasion, metastasis and recurrence. Microvessel density (MVD) and microvessel area (MVA) measured by immunohistochemical technique have been considered as the criteria for evaluating tumor angiogenesis, which reflect the intensity of blood vessels, the area of endothelial lumen and the blood volume for tumor perfusion [7, 8]. Nevertheless, studies on different types of RCC, including papillary and clear cell RCC, demonstrate that MVD and MVA are negatively correlated with survival rate, directly correlated or of no significant difference [9]. The reasons for these contradictory findings are not completely explicit. CT and MR perfusion imaging, as well as intravoxel incoherent motion (IVIM) model for diffusion weighted, were once perceived to be ideal for preoperative noninvasive evaluation of tumor angiogenesis [10]. However, they are limited in clinical application due to the large radiation dose, long examination time and other factors.

In recent years, spectral imaging has been introduced as a new application of dual energy CT and an advanced CT scanning technology [11]. Energetic-spectrum computed tomography (spectral CT) is able to provide iodinebased material decomposition image and quantitatively analyze the iodine concentration (IC) of normal tissues and lesions in enhanced images. IC possesses a certain value in the imaging diagnosis, pathological grading and differential diagnosis of RCC [12, 13]. However, only a few studies pay close attention to the relationship between IC derived from spectral CT and RCC angiogenesis. Therefore, it is of vital importance to explore the expression of IC and WC on the angiogenesis of tumor. This study aims to analyze the relationship between IC and WC with RCC angiogenesis.

## Materials and methods

### Participants

The study was approved by the Institutional Ethics Committee of the First Hospital of Jilin University, Changchun, China. Written informed consent was obtained from related patients by email or letter. We retrospectively reviewed the Picture Archiving and Communication System for renal cell carcinoma patients who had undergone renal CT plain scan plus three-phase enhancement before surgery from January 2017 to December 2019. The inclusion criteria were as follows: 1) patients aged between 18 and 80 years old; 2) renal CT plain scan plus three-phase enhancement before surgery; 3) patients who had undergone CT performed according to our standard protocol; 4) patients who had received standard nephrectomy; 5) The interval between CT scan and surgery was no more than 2 weeks. When evaluating tumor angiogenesis index, in order to ensure the consistency of baseline level, the following inclusion criteria should also be met: 1) RCCs were all single without distant metastasis; 2) The maximum diameter of RCC was between 3 and 5 cm.

The exclusion criteria were as follows: 1) Imaging data with poor quality due to body movements or other artifacts were excluded; 2) Patients who were found to be allergic to iodine contrast agent before enhanced CT examination were excluded; 3) Patients who are RCC complicated with hemorrhage were excluded; 4) Cystic RCC and RCC with large cystic lesions were excluded to ensure the same baseline level of tumor angiogenesis index.

### Energy Spectrum CT imaging

Renal three-phase enhancement CT was performed on a Revolution GSI CT scanner (GE Healthcare). Scanning range was conducted from “diaphragmatic apex to lower pole of kidney” with supine position. The CT scan was conducted with the following parameters: tube voltage, automatically selected by machine according to the patient’s weight; tube current, 485 mA; pitch, 0.984; field of view (FOV), medium; image matrix, 512 × 512; rotation speed, 0.6; slice thickness/gap, 5/5 mm. Renal three-phase enhancement scan: high pressure syringe was utilized to inject non-ionic contrast agent iohexol or iopatol through elbow vein, with the dosage of 1.0–1.5 ml/kg and the speed of 3.5 ml/s. The cortical phase scanning was performed at 25 s after injection, renal parenchymal phase scanning was performed at (60 ± 5) s, and excretion phase scanning was performed at (180 ± 30) s after injection. During the scanning, the patient was required to hold his breath after deep inspiration to avoid motion artifacts affecting the image quality.

### Acquisition of iodine and water concentration

The iodine and water concentration of renal three-phase enhancement CT were measured in Picture Archiving and Communication Systems (GE, Version AW4.6, USA). The IC of each tumor demands to take the average of six values on the decomposition images for iodine (120kev). These six values include four values in the axial maximum plane and two values in the coronal maximum plane. The four measurement values of the maximum axial plane include the measurement values of the directions in front, back, left and right. The region of interest (ROI) of these four directions should not cross each other and keep as far as possible to avoid obvious necrosis. It’s required to keep the area of ROI consistent. The WC of each tumor was obtained on the water equivalent images in the same way as above. The iodine concentration in the aorta (IC_ao_) at the same slice of the lesion was obtained. Normalized iodine concentration (NIC) was normalized to the IC_ao_ using the formula NIC = IC/IC_ao_. Likewise, the normalized WC (NWC) was obtained with the formula NWC = WC/WC_ao_. (N)WCD_1_/ICD_1_ = The difference of (N)WC/IC between renal cortical phase and parenchymal phase. (N)WCD_2_/ICD_2_ = The difference of (N)WC/IC between renal parenchymal phase and excretory phase. (N)WCD_3_/ICD_3_ = The difference of (N) WC /IC between renal cortical phase and excretory phase. The above measurements were conducted independently by three radiologists with more than 10 years working experience. All the values were taken as the average of the three radiologists.

### Interpretation of clinicopathological results

According to the electronic medical records, the pathologic findings such as tumor types, tumor classifications, and stage of tumor were assessed. Distant metastasis was defined by imaging examinations and clinical follow-up [14].

### Immunohistochemical analysis

Immunohistochemistry was used to stain tumor microvessels. The first antibody was CD34. The thickness of paraffin fixed sections was 5 μ m, which was dewaxed and hydrated. Microwave antigen repair, 2% citrate buffer, PH 6.0, 15mins. After the first antibody was dripped, the slices were incubated in a wet box at 4 °C overnight. After the universal second antibody was dripped, the slices were placed in a wet box and incubated at 37 °C for 30 mins. The slices were washed with TBS (0.01 mol / L, pH 7.4) for 3 times. After DAB staining, the nuclei were stained with hematoxylin. Finally,the slide was sealed.

### Analysis of tumor microvessels

Five sections were offered for per patient, the average value of microvessel density, area and grading was taken from the five sections. As long as no less than one slice has characteristic blood vessels, the characteristic blood vessels can be defined as positive.

**Fig. 1:**
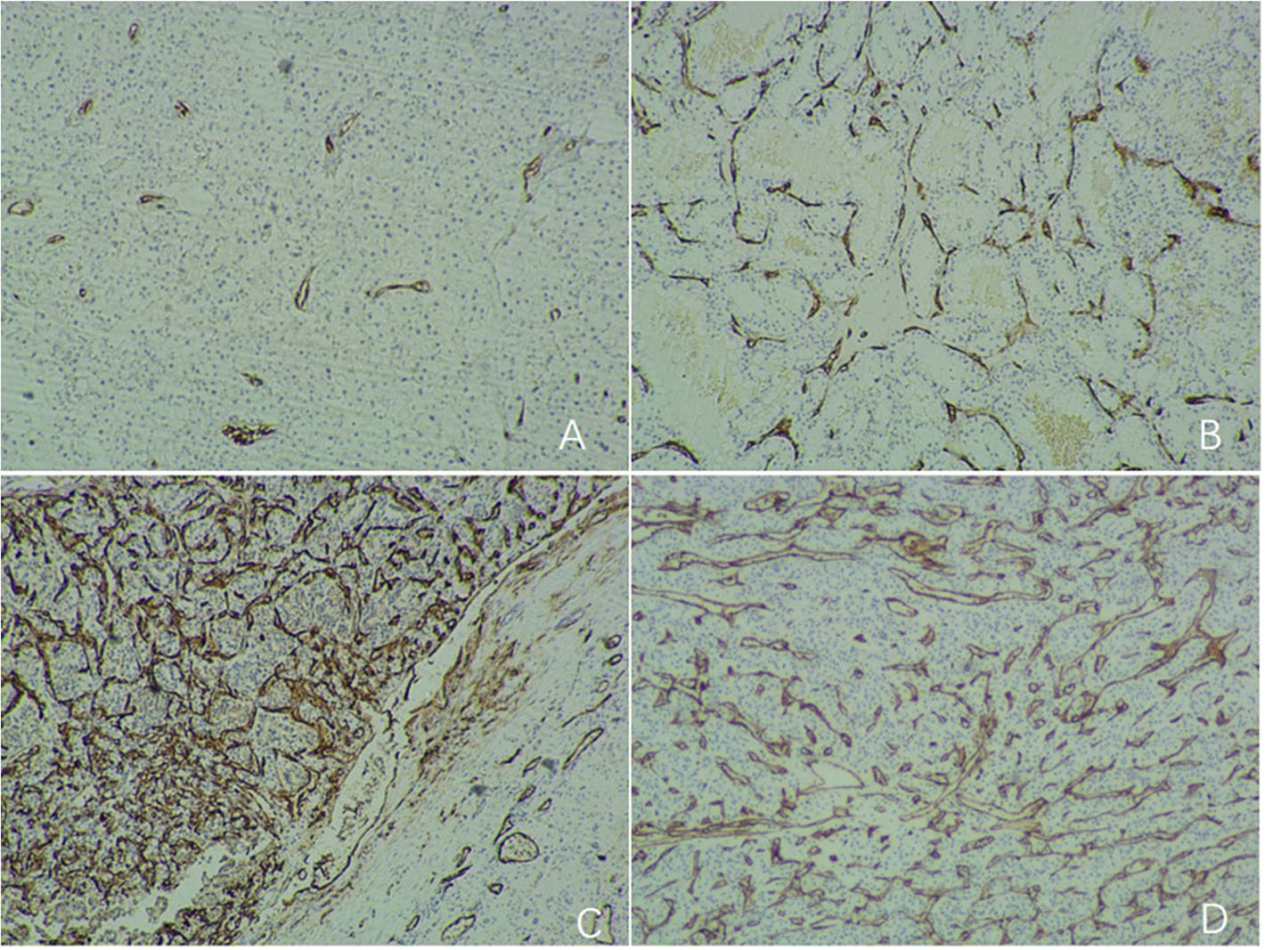
CD34 staining (× 40) show punctiform microvessels (**A**), linear microvessel (**B**), annular microvessels (**C**) and strip type microvessels (**D**). The cytoplasm of tumor vascular endothelial cells is extensively stained with CD34

1) Mean microvessel density (MVD): After staining, the high vascular density area was found under low power microscope. The top five regions with the higher vascular density in each slice were selected. The number of microvessels in each field of vision was counted. The average value of the five regions is calculated as mean MVD. When counting, only a single endothelial cell or cell cluster is counted, otherwise the vascular lumen is not counted as microvessel.

2) Microvascular area (MVA): Percentage of microvessel area in tumor tissue area.

3) Microvascular classification: Punctiform microvessels displayed that the microvessels were divided by tumor cells and distributed in punctiform shape, with no communication between the microvessels. The linear microvessel manifest that there were punctate microvessels on both sides of the microvessel, with the middle long and narrow, which was linear. The annular microvessels are characterized by thick microvessels, which encircle the tumor cells in an island shape and communicate with each other. Strip type microvessels showed thick microvessels and paralleled to tumor trabeculae, communicating with each other as well as showing a thick network. The tumor microvascular classification was semi quantified: the punctiform type was grade I, the linear type was grade II, and the annular or strip type was grade III (Fig. 1). The microvascular grade of the tumor was in line with the majority of the grading results in any 10 high-power fields.

4) Analysis of special vascular structure: Record whether there are vascular lakes, vascular trees (One main vessel accompanied by branch vessels), thick muscular vessels or vessels with diameter more than 50 μm appearing.

### Statistical analysis

Pearson’s or Spearman’s correlation test was applied to test the correlation between WC and IC values (mean WC, mean IC, random WC, random IC, mean NWC and mean NIC in renal cortical phase, parenchymal phase and excretory phase; WCD, NWCD, DIC and NICD parameters) with microvessel parameters (MVD, MVA, specific microvascular, microvascular grading, vessel diameter and tumor diameter). All statistical analyses were performed using SPSS 21.0 (SPSS). *P* < 0.05 was considered statistically significant.

## Results

### Demographic and relevant characteristics

A total of 32 patients with RCC were included in the study. The nuclear grade of RCC was positively correlated with MVD (Spearman rank correlation coefficients, r = 0.872, *p* < 0.001). Table 1 shows their demographic, pathological microvascular data and values of iodine and water concentration in three phase enhancement. In this study, RCC affected men more frequently than women. Most of the measurement data is in line with normal distribution, only a few (MVD, NMWC in renal cortical phase, NMIC in renal excretory phase, RIC in renal cortical phase, ICD_1_, ICD_2_, NICD_2_, NICD_3_, NWCD_2_ and NWCD_3_) are skew distribution.

**Table 1:** Demographic and clinical characteristics

Variable	Patients (*n* = 32)
Age (years)	53.53 ± 8.15
Male/Female	25 (78.1%)/7 (21.9%)
MWC/MNWC (mg/ml)	
Renal cortical phase	95.61 ± 33.89/0.42 (0.31–0.47)
Renal parenchymal phase	82.74 ± 20.54/0.39 ± 0.15
Renal excretory phase	65.37 ± 12.21/0.78 ± 0.17
RWC/RNWC (mg/ml)	
Renal cortical phase	95.53 ± 44.48/0.39 ± 0.23
Renal parenchymal phase	85.06 ± 23.07/0.73 ± 0.21
Renal excretory phase	65.26 ± 14.14/0.75 ± 0.18
MIC/MNIC (mg/ml)	
Renal cortical phase	28.99 ± 14.52/0.33 ± 0.14
Renal parenchymal phase	24.65 ± 8.37/0.71 ± 0.23
Renal excretory phase	17.09 ± 6.60/0.91 (0.78–1.05)
RIC/RNIC (mg/ml)	
Renal cortical phase	27.00 (19.50–34.25)/ 0.32 ± 0.16
Renal parenchymal phase	24.16 ± 8.86/0.70 ± 0.27
Renal excretory phase	16.95 ± 5.49/0.79 ± 0.27
WCD_1_/NWCD_1_ (mg/ml)	12.87 ± 21.26/−0.30 ± 0.13
WCD_2_/NWCD_2_ (mg/ml)	30.24 ± 27.82/− 0.40[− 0.46-(− 0.30)]
WCD_3_/NWCD_3_ (mg/ml)	17.37 ± 13.38/− 0.12(− 0.22–0.02)
ICD_1_/NICD_1_ (mg/ml)	4.14(−2.91–8.17)/ -0.388 ± 0.15
ICD_2_/NICD_2_ (mg/ml)	13.17 (2.18–16.34)/ -0.56[− 0.75-(− 0.42)]
ICD_3_/NICD_3_ (mg/ml)	7.56 ± 4.92/− 0.21[− 0.36-(− 0.05)]
Microvascular grading	
II	15 (46.9%)
III	17 (53.1%)
MVD	26.00 (15.00–43.75)
MVA(%)	16.16 ± 8.98
Microvascular diameter (μm)	20.63 ± 9.73
Diameter of analyzed tumor tissue (μm)	55.19 ± 19.15
Microvascular diameter/ Tumor diameter (%)	40.98 ± 19.66

Data are expressed as mean ± standard deviation, median (interquartile range) or n (%). *MVD* microvessel density, *MVA* microvessel area. *(N)WCD*_*1*_*/ICD*_*1*_ The difference of (N)WC/IC between renal cortical phase and parenchymal phase. *(N)WCD*_*2*_*/ICD*_*2*_ The difference of (N)WC/IC between renal parenchymal phase and excretory phase. *(N)WCD*_*3*_*/ICD*_*3*_ The difference of (N) WC /IC between renal cortical phase and excretory phase

### Imaging data

Correlation between microvessel parameters and WC/IC in renal cortical phase.

The correlations between the WC/IC in renal cortical phase and microvessel parameters were analyzed. The mean WC, mean IC, mean NWC, mean NIC, random IC and random NIC from renal cortical phase scanning correlated positively with the MVD of RCC (Spearman rank correlation coefficients, all *p* < 0.05), as shown in Table 2. In the same scanning phase, the correlations between the mean NIC and mean NWC with MVD were better than those between the mean IC and mean WC with MVD. Moreover, for the same phase, the correlations between the mean IC and mean NIC with MVD were stronger than those between the random IC and random NIC with MVD. The mean WC, mean IC, mean NWC, mean NIC, random IC and random NIC from renal cortical phase scanning correlated positively with the MVA of RCC (Pearson or Spearman rank correlation coefficients, all *p* < 0.05), as displayed in Table 2. In the same scanning phase, the correlations between the mean IC and mean NWC with MVA were better than those between the mean NIC and mean WC with MVA. Besides, for the same phase, the correlations between the mean IC and mean NIC with MVA were stronger than those between the random IC and random NIC with MVA. The mean NWC, mean NIC and random NIC had moderate positive correlation with microvascular grading (Spearman rank correlation coefficients, all *p* < 0.05), and the correlation between the mean NIC and microvascular grading was stronger than that between the mean NWC with microvascular grading.

**Table 2:** Linear regression results evaluating the correlation of microvessel parameters and WC/IC in renal cortical phase

Microvessel Parameters	Mean WC		Mean IC		Mean NWC		Mean NIC		Random WC		Random IC		Random NWC		Random NIC	
	r	*P* value	r	*P* value	r	P value	r	*P* value	r	*P* value	r	*P* value	r	*P* value	r	*P* value
MVD	0.362	0.042*	0.421	0.016*	0.524	0.002*	0.484	0.005*	0.151	0.409	0.413	0.019*	0.287	0.112	0.383	0.031*
MVA	0.494	0.004*	0.477	0.006*	0.520	0.002*	0.469	0.007*	0.236	0.149	0.370	0.037*	0.293	0.104	0.357	0.045*
Specific microvascular	0.121	0.509	0.139	0.450	0.259	0.153	0.259	0.157	0.032	0.863	0.160	0.381	0.246	0.175	0.185	0.311
Microvascular grading	0.214	0.240	0.281	0.119	0.448	0.010*	0.479	0.006*	−0.031	0.868	0.285	0.114	0.156	0.393	0.367	0.039*
Vessel diameter	0.158	0.338	0.127	0.487	0.259	0.152	0.265	0.201	0.046	0.802	0.192	0.292	0.103	0.576	0.048	0.796
Tumor diameter	−0.242	0.182	− 0.242	0.181	− 0.013	0.943	− 0.127	0.490	−0.190	0.298	−0.307	0.087	−0.075	0.684	−0.170	0.353
Vessel diameter/ Tumor diameter	0.161	0.380	0.156	0.393	0.142	0.439	0.107	0.560	0.037	0.840	0.321	0.073	0.063	0.731	0.092	0.617

*means that *P*<0.05, with statistical significance. Mean (N)WC/IC was the average of the six measurements mentioned in the method. Random (N)WC/IC was the random of the six measurements mentioned in the method. Most of the Mean and Random (N)WC/ICs showed moderate correlation with MVD, MVA and microvascular grading

### Correlation between microvascular parameters and WC/IC in renal parenchymal phase

The relationships between microvascular parameters and WC/IC of renal parenchymal phase among the 32 patients enrolled in the study were presented in Table 3. The mean WC and mean NWC correlated positively with the MVA of RCC (Pearson rank correlation coefficients, both *p* < 0.05). Besides, the correlation between the mean NWC and MVA was slightly stronger than that between the mean WC with MVA. At the same time, the mean NWC was also discovered to be positively correlated with microvascular grading (Pearson rank correlation coefficients, *p* < 0.05).

**Table 3:** Multiple linear regression results evaluating the correlation of microvessel parameters and WC/IC in renal parenchymal phase

Microvessel Parameters	Mean WC		Mesn IC		Mean NWC		Mean NIC		Random WC		Random IC		Random NWC		Random NIC	
	r	*P* value	r	*P* value	r	*P* value	r	*P* value	r	*P* value	r	*P* value	r	*P* value	r	*P* value
MVD	0.158	0.387	0.221	0.225	0.284	0.115	0.227	0.211	0.120	0.513	0.118	0.520	0.179	0.326	0.130	0.479
MVA	0.355	0.046*	0.336	0.060	0.363	0.041*	0.279	0.122	0.274	0.130	0.306	0.088	0.289	0.108	0.247	0.173
Specific microvascular	0.014	0.938	−0.023	0.900	0.055	0.765	−0.072	0.695	0.064	0.730	− 0.169	0.355	0.061	0.742	− 0.095	0.604
Microvascular grading	0.200	0.272	0.187	0.307	0.390	0.027*	0.200	0.272	0.268	0.138	0.163	0.373	0.326	0.069	0.234	0.197
Vessel diameter	−0.022	0.906	−0.034	0.853	0.016	0.932	−0.037	0.839	−0.150	0.413	−0.158	0.388	−0.086	0.638	−0.145	0.429
Tumor diameter	−0.122	0.508	− 0.175	0.339	0.041	0.822	−0.117	0.522	−0.083	0.650	−0.204	0.263	0.056	0.761	−0.104	0.573
Vessel diameter/ Tumor diameter	−0.046	0.801	−0.008	0.967	−0.060	0.745	0.007	0.972	−0.167	0.360	−0.074	0.686	−0.143	0.433	−0.084	0.649

*means that *P*<0.05, with statistical significance. Mean WC and NWC showed moderate correlation with MVA

### Correlation between microvascular parameters and WC/IC in renal excretory phase

The correlations between the WC/IC indexes in renal excretory phase and microvascular parameters of RCC were demonstrated in Table 4. No significant correlations between all WC / IC derived indicators and microvascular parameters (Pearson or Spearman rank correlation coefficients, all *p* > 0.05) were found.

**Table 4:** Multiple linear regression results evaluating the correlation of microvessel parameters and WC/IC in renal excretory phase

Microvessel Parameters	Mean WC		Mesn IC		Mean NWC		Mean NIC		Random WC		Random IC		Random NWC		Random NIC	
	r	*P* value	r	*P* value	r	*P* value	r	*P* value	r	*P* value	r	*P* value	r	*P* value	r	*P* value
MVD	−0.095	0.606	0.215	0.237	−0.035	0.850	0.137	0.455	−0.042	0.819	0.272	0.132	−0.026	0.889	0.137	0.455
MVA	0.059	0.749	0.186	0.308	0.019	0.917	−0.007	0.971	0.092	0.618	0.333	0.162	0.053	0.779	−0.045	0.806
Specific microvascular	−0.156	0.394	−0.032	0.863	−0.298	0.098	−0.159	0.385	0.001	0.994	0.023	0.900	−0.084	0.648	0.159	0.385
Microvascular grading	−0.112	0.542	0.132	0.471	−0.261	0.148	−0.193	0.285	0.024	0.897	0.156	0.393	−0.170	0.353	−0.193	0.289
Vessel diameter	−0.303	0.092	−0.062	0.735	−0.246	0.175	0.137	0.455	−0.245	0.177	0.068	0.710	−0.207	0.255	0.151	0.408
Tumor diameter	−0.308	0.086	−0.164	0.369	−0.213	0.242	−0.095	0.603	−0.292	0.105	--0.287	0.111	−0.223	0.220	−0.122	0.505
Vessel diameter/ Tumor diameter	−0.135	0.462	−0.006	0.975	−0.054	0.767	0.236	0.194	−0.124	0.500	0.177	0.332	−0.046	0.803	0.312	0.082

None of the Mean (N)WC/IC and Random (N)WC/IC showed correlation microvascular parameters

### Correlation between microvascular parameters and DWC

**Fig. 2:**
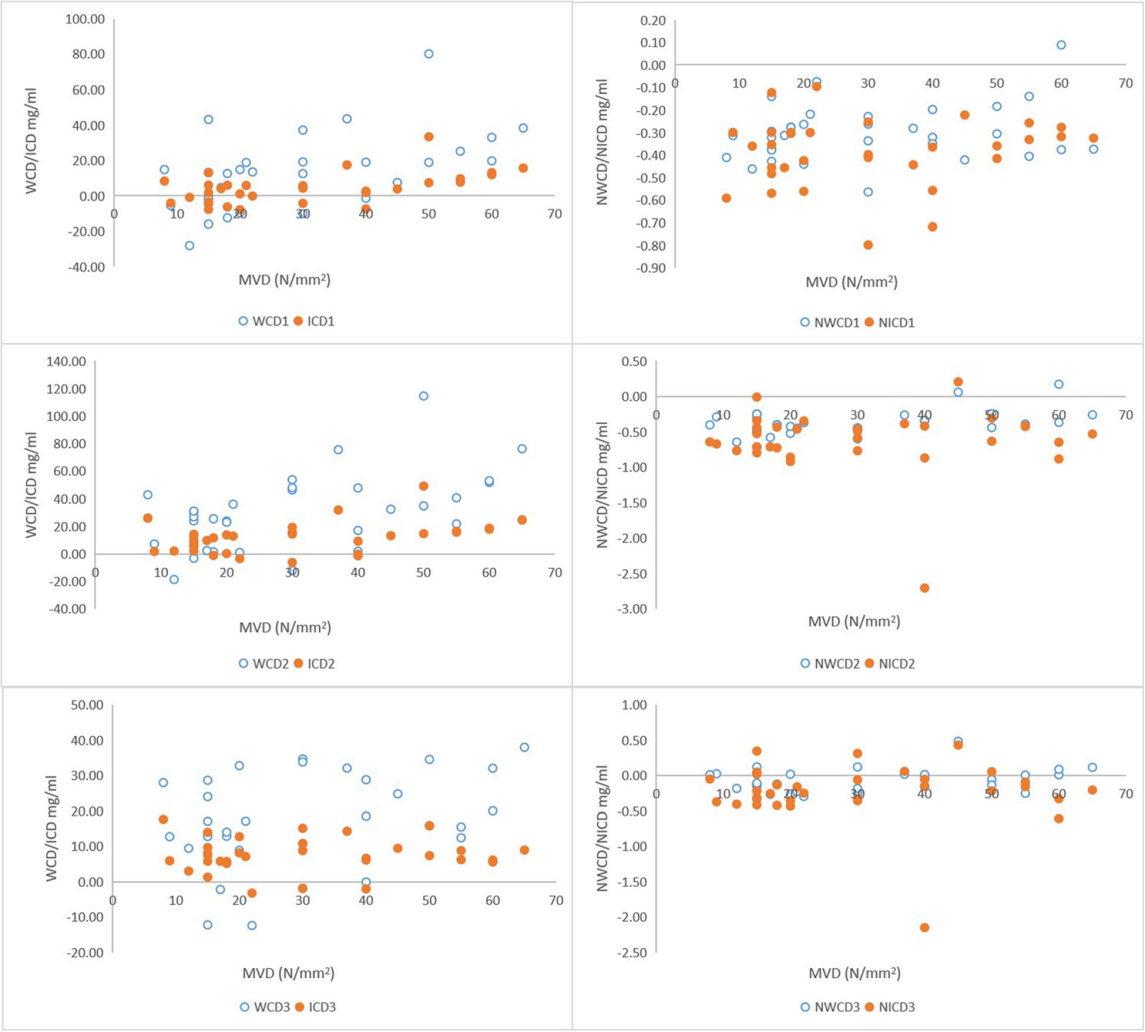
The relationship between WCD, ICD and MVD in thirty-two renal cell carcinoma patients are exhibited. The relationship between NWCD, NICD and MVD are also shown for comparison. Several of the difference of ICD and WCD between cortical phase and medullary phase, including standardized difference, show a positive correlation with MVD. (N)WCD_1_/ICD_1_ = The difference of (N)WC/IC between renal cortical phase and parenchymal phase. (N)WCD_2_/ICD_2_ = The difference of (N)WC/IC between renal parenchymal phase and excretory phase. (N)WCD_3_/ICD_3_ = The difference of (N) WC /IC between renal cortical phase and excretory phase

The correlations between WCD indicators and microvessel parameters were exhibited in Table 5, Fig. 2A, C and E. The WCD_1_, WCD_2_, and NWCD_2_ correlated positively with the MVD of RCC (Spearman rank correlation coefficients, all *p* < 0.05). And the correlation between WCD_1_ and MVD was higher than that between WCD_2_ and MVD. Moreover, the correlation between the NWCD_2_ was lower than that between WCD_2_ and MVD.

**Table 5:** Multiple linear regression results evaluating the correlation of microvessel parameters and WCD

Microvessel Parameters	WCD_1_		NWCD_1_		WCD_2_		NWCD_2_		WCD_3_		NWCD_3_	
	r	*P* value	r	*P* value	r	*P* value	r	*P* value	r	*P* value	r	*P* value
MVD	0.533	0.002*	0.197	0.281	0.509	0.003*	0.366	0.040*	0.333	0.063	0.184	0.313
MVA	0.445	0.011*	0.091	0.619	0.576	0.001*	0.401	0.023*	0.491	0.004*	0.413	0.019*
Specific microvascular	0.216	0.234	0.233	0.200	0.216	0.234	0.441	0.012	0.266	0.142	0.202	0.266
Microvascular grading	0.220	0.225	−0.027	0.883	0.268	0.138	0.520	0.002*	0.397	0.025*	0.476	0.006*
Vessel diameter	0.273	0.130	0.249	0.169	0.326	0.069	0.432	0.013	0.243	0.180	0.271	0.134
Tumor diameter	−0.268	0.138	− 0.158	0.386	−0.159	0.383	0.177	0.332	0.095	0.606	0.388	0.028*
Vessel diameter/ Tumor diameter	0.301	0.094	0.309	0.085	0.255	0.159	0.126	0.490	0.052	0.779	−0.067	0.714

*means that *P*<0.05, with statistical significance. *(N)WCD*_*1*_ The difference of (N) WC between renal cortical phase and parenchymal phase. *(N)WCD*_*2*_ The difference of (N) WC between renal parenchymal phase and excretory phase. *(N)WCD*_*3*_ The difference of (N) WC between renal cortical phase and excretory phase. Several of the (N) WCDs showed moderate correlation with MVD, MVA and microvascular grading

For MVA, the WCD_1_, WCD_2_, NWCD_2_, WCD_3_, and NWCD_3_ correlated positively with the MVA of RCC (Pearson or Spearman rank correlation coefficients, all *p* < 0.05). According to the strength of association, the order is as follows: WCD_2_,>WCD_3_ > WCD_1_. The correlations between the WCD_2_ and WCD_3_ with MVA were stronger than those between the NWCD_2_ and NWCD_3_ with MVA.

The WCD_3_, NWCD_2_, and NWCD_3_ correlated positively with the microvascular grading of RCC (Spearman rank correlation coefficients, all *p* < 0.05). The correlation between the WCD_3_ and microvascular grading was weaker than that between the NWCD_3_ and microvascular grading. What’s more, DNWC_3_ was moderately correlated with the vessel diameter (Spearman rank correlation coefficients, *p* < 0.05).

### Correlation between microvascular parameters and DIC

The correlations between DIC indicators and microvessel parameters were displayed in Table 6, Fig. 2B, D and F. The DIC_1_ correlated positively with the MVD of RCC (Spearman rank correlation coefficients, *p* < 0.05). The NICD_2_ and NICD_3_ correlated positively with the MVD of RCC (Spearman rank correlation coefficients, both *p* < 0.05). The correlation between the NICD_2_ and microvascular grading (r = 0.397) was stronger than that between the NICD_3_ and microvascular grading (r = 0.383).

**Table 6:** Multiple linear regression results evaluating the correlation of microvessel parameters and ICD

Microvessel Parameters	ICD_1_		NICD_1_		DICD_2_		NICD_2_		ICD_3_		NICD_3_	
	r	*P* value	r	*P* value	r	*P* value	r	*P* value	r	*P* value	r	*P* value
MVD	0.473	0.006*	0.172	0.347	0.408	0.020	0.111	0.546	0.060	0.746	0.067	0.714
MVA	0.398	0.024	0.017	0.925	0.540	0.001	0.225	0.215	0.427	0.015	0.243	0.180
Specific microvascular	0.225	0.215	0.317	0.077	0.153	0.403	0.199	0.274	−0.019	0.919	0.094	0.609
Microvascular grading	0.217	0.233	0.187	0.305	0.234	0.197	0.397	0.024*	0.190	0.298	0.383	0.030*
Vessel diameter	0.211	0.246	0.162	0.375	0.227	0.212	−0.035	0.849	0.025	0.890	−0.084	0.648
Tumor diameter	−0.210	0.248	0.068	0.710	−0.149	0.415	−0.052	0.777	−0.077	0.675	−0.034	0.853
Vessel diameter/ Tumor diameter	0.300	0.096	0.077	0.677	0.256	0.157	−0.122	0.505	−0.005	0.976	−0.178	0.330

*means that *P*<0.05, with statistical significance. *(N)ICD*_*1*_ The difference of (N) IC between renal cortical phase and parenchymal phase. *(N)ICD*_*2*_ The difference of (N) IC between renal parenchymal phase and excretory phase. *(N)ICD*_*3*_ The difference of (N) IC between renal cortical phase and excretory phase. ICD_1_ showed moderate correlation with MVD. NICD2 and NICD3 showed weak correlation with microvascular grading

## Discussion

The incidence rate of RCC has raised in recent years, ranking the seventh in the most frequently diagnosed malignancies [15]. Renal cell carcinoma is one of the most hyper-vascularized tumors. High levels of HIF-1 and HIF-2 mediate the production of vascular endothelial growth factor (VEGF), which explains the high vascularization of RCC. Metabolic disorders and angiogenesis caused by hypoxia are the microenvironment basis of renal cell carcinoma metastasis. Angiogenesis is crucial for tumor growth and metastasis. The new microvessels in the tumor are the main sites for tumor cells to enter circulation. The 5-year survival rate of metastatic renal cell carcinoma was less than 10% [16].

CT and MR perfusion are utilized to assess tumor angiogenesis. Nevertheless, perfusion parameters are not correlated to microvascular angiogenesis. Perfusion index (PI) reflects the ability of blood perfusion. The high PI of tumor tissue is not only related to the number of microvessels, but also related to that of large vessels. It may be the reason that microvascular parameters such as MVD sometimes don’t have obvious correlation with CTP and MTP parameters. Nevertheless, due to the limitation of exam methods, the above opinions need to be supplemented by new means. The minimum voltage (80kvp) and the maximum voltage (140kvp) are usually used to achieve the maximum energy separation to distinguish different substances. Spectral CT scan quantitatively analyze the iodine and water density of RCC by generating decomposition image Spectral CT can be combined with iodine contrast to calculate iodine concentration in lesions. It can be used to evaluate the microvascular parameters of various solid tumors including MVD, MVA, etc.

**Fig. 3:**
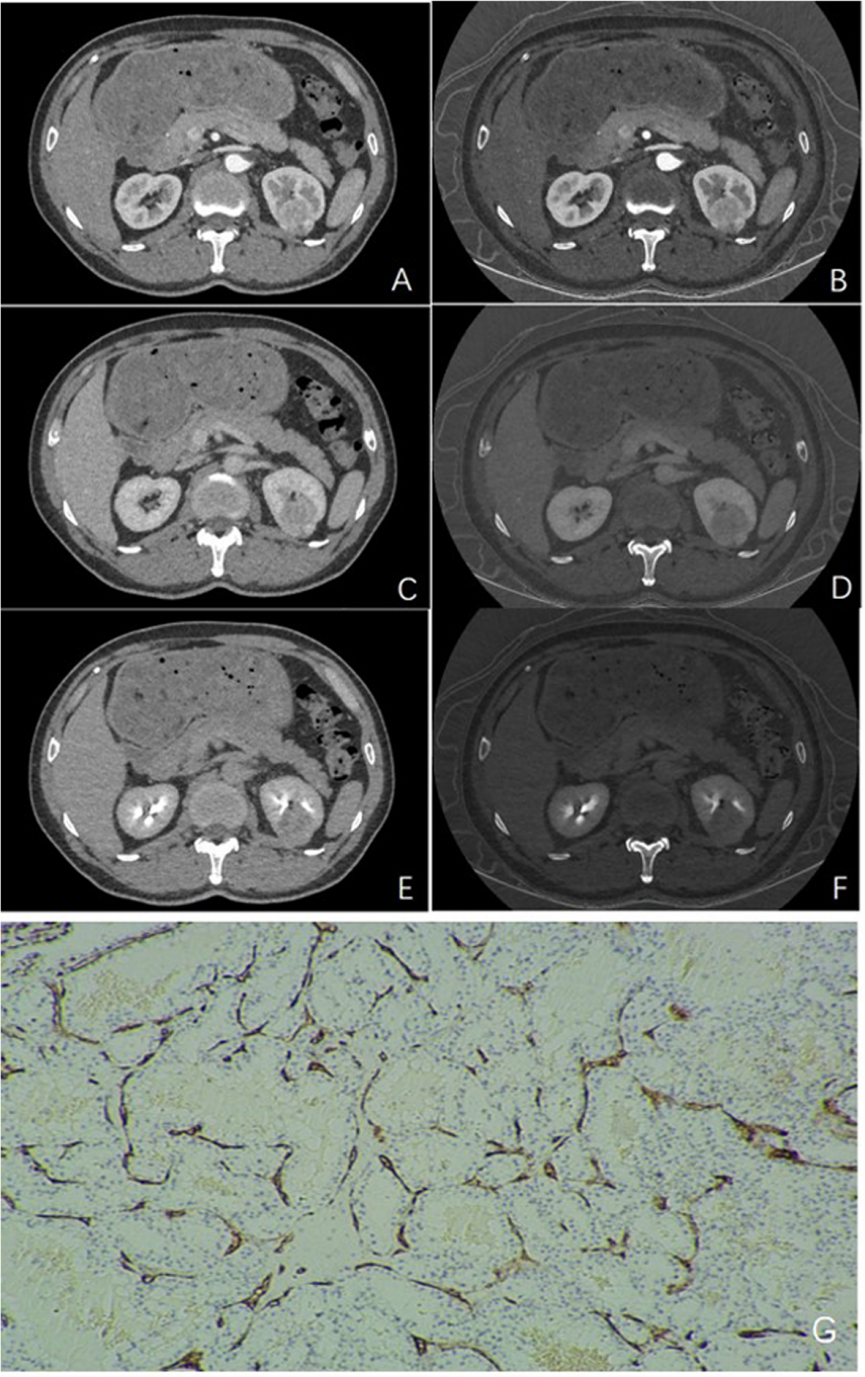
Images from a 58-year-old man with a 3.2-cm single renal cell carcinoma. The enhancement of left renal tumor was obvious in renal cortical phase, but slightly decreased in parenchymal phase and excretory phase (**A**, **C** and **E**). **B**, **D** and **F** show the corresponding iodine concentration of lesion. Immunohistochemical staining shows CD34-positive microvessel density. The positively stained endothelial cell or endothelial cell cluster which appears obviously brown is counted as microvessel. The mean microvascular count is 55 (**G**, × 40)

In our study, iodine and water were utilized as the bascs material pair in material decomposition and were also used to measure IC and WC in kidney. The density of iodine and water in tumor can be quantitatively analyzed by spectral CT. the MVD, MVA and other microvascular parameters were evaluated on the basis of macroscopic imaging [17]. Only the mean IC, mean WC, mean NWC and mean NIC of renal cortex phase were positively correlated with MVD, suggesting that tumor cells were metabolized vigorously and blood supply was abundant, while there were no significant correlations between the above parameters and MVD in the other two phases (Fig. 3). In the other two phases, the correlation between IC value and microvascular parameters is lower than that of cortical phase, which may be related to the blood flow characteristics of the kidney itself. In addition, RCC is abundant in blood supply, so the IC of cortical phase can reflect the real situation of microvessels best. Some studies have demonstrated that the NIC value of enhanced CT scan was positively correlated with MVD [18]. A retrospective study of 60 patients with lung cancer showed that MVD was positively correlated with IC, ICD and NIC (range 0.581–0.800, all < 0.001). Similar to our study, the correlation between IC and ICD and MVD is better than that between NIC and MVD. There was a certain amount of iodine in tumor tissue without injection of CT contrast agent. In addition, a mixture of different substances exist in the tissue, and the mixture of the two basic materials can be quantitatively measured by spectral CT. ICD can eliminate these confounding factors better. NIC usually refers to the ratio of IC in tumor to IC in thoracic aorta at the same level. It can be seen that NIC may also be affected by the degree of aortic enhancement when the blood supply of the subjects is different, which may also make NIC deviate from the actual IC of the lesion [19, 20]. It is believed that although angiogenesis is related to microvascular density, it does not necessarily lead to high blood flow, because the increased interstitial fluid pressure of immature and leaky new vessels may reduce blood flow and lead to tissue hypoxia [21].

IC mainly reflects the blood supply of microvascular RCC in the renal cortical phase, while IC may indicate the flow and retention of blood supply in the renal medullary phase. It is worth noting that cortical phase enhancement is more prominent in RCC, suggesting that cortical phase IC is more useful for the detection of angiogenesis in RCC. The IC of renal cortical phase image reflects the density of tumor capillaries, and the difference of renal cortical phase and medullary phase image reflects the clearance of tumor capillaries and tumor matrix [22]. In renal parenchymal phase, mean IC, mean NIC, random IC and random NIC had no prominet correlation with microvascular parameters. Iodine is the main component of CT contrast agent, so IC can effectively evaluate the blood supply of tumor. Given that renal medulla is located in the deep layer of renal cortex with few blood vessels, and account for the vast majority of renal parenchyma,. it is reasonable to explain that the indicators derived from IC are not related to microvascular conditions.

NIC and NWC were calculated in order to normalize the differences in individual circulation between patients. We discovered that the correlation between NIC and NWC with microvascular parameters was not significantly better than that of IC and WC. NIC and NWC did not show more stable as an index, which was slightly inconsistent with previous studies [23]. Some studies also implied that NIC depends on the extent of lesion and aortic enhancement, and may make NIC deviate from the actual IC of lesion. This conclusion is similar to our searching result [19]. Therefore, in view of our findings in this study, IC may be a better indicator of tumor angiogenesis than NIC. When the RCC microvessels are relatively mature and the blood flow is fast, the contrast medium is easy to enter. In tumor tissue, when the differentiation degree of tumor is low, RCC grows fast, then the neovascularization structure is incomplete and unevenly distributed. These lesions show abnormal hemodynamics, resulting in uneven distribution of blood supply [24]. Therefore, in cortical and medullary phase, especially in cortical phase, NIC and NWC correlated with microvascular grading.

It has been shown that the average iodine density of larger tumors is significantly lower than that of the smaller ones [25], which is inconsistent with the results of our study. There were no significant correlations between derived parameters of IC and WC with tumor diameter except NWCD_3_. That is to say, in this study, the larger the tumor, the faster the water excretion from the cortical phase to the renal pelvis secretory phase. The present study showed that among the difference of cortical phase,medullary phase as well as medullary phase, the correlation between WC derived indexes and angiogenesis seems to be more significant than that of IC derived indexes. It is becuase the water outflow from tumor tissue is more easily affected by the changes of microvascular structure with the increase of microvascular area and the complexity of microvascular structure [26]. In addition, the molecular weight of water is smaller than that of iodine compound, and the flow rate is faster. The change of WC in different phases can represent the situation of microvessels better .

Several limiting factors must be considered in this study. Firstly, the study included a relatively small number of RCC patients. We believe that our research may encourage future research, and a large multicenter study will be desirable. Secondly, the relationship between the prognosis of these RCC patients and IC and WC is worth exploring. Moreover, although pathologists and radiologists are particularly careful in sampling, it is difficult to match histopathological sections with imaging sections accurately. RCCs may show different characteristics in different perspects of view. In clinical practice, the ROI is delineated at the maximum level of axial and coronal tumor, also the average value of each measurement is calculated as the final result to minimize the systemic errors. Although strict inclusion and exclusion criteria were established, selection bias still existed due to the retrospective study. Further studies will be carried out with expanded samples. Finally, the correlation between WC and IC parameters on microvascular status is still in research stage, and it needs to be further verified in the follow-up study before applied in clinical practice.

## Conclusion

ICD and WCD of spectral CT have potential for evaluating RCC microvascular angiogenesis. MVD, MVA and microvascular grade showed moderate positive correlation with ICD and WCD. ICD displayed more relevant than that of WCD. The parameters of renal cortical phase were the best in three phases. NICD and NWCD demonstrated stronger correlation with microvascular parameters than that of ICD and WCD.

## Data Availability

The datasets generated and/or analyzed during the current study are not publicly available due to the Personal Information Protection Act, but are available from the corresponding author upon reasonable request.

## Abbreviations

ccRCC: Clear cell renal cell carcinoma
IC: Iodine concentration
ICD: Iodine concentration difference
IVIM: Intravoxel incoherent motion
MVA: Microvascular area
MVD: Microvascular density
MIC: Mean iodine concentration
MNIC: Mean normalized iodine concentration
MNWC: Mean normalized water concentration
MWC: Mean water concentration
PI: Perfusion index
RCC: Renal cell carcinoma
RIC: Random iodine concentration
ROI: Region of interest
RWC: Random water concentration
NRIC: Normalized random iodine concentration
NRWC: Normalized random water concentration
WC: Water concentration
WCD: Water concentration difference
